# Electric-field-driven magnetization switching and nonlinear magnetoelasticity in Au/FeCo/MgO heterostructures

**DOI:** 10.1038/srep29815

**Published:** 2016-07-18

**Authors:** P. V. Ong, Nicholas Kioussis, P. Khalili Amiri, K. L. Wang

**Affiliations:** 1 Department of Physics and Astronomy, California State University Northridge, Northridge, California 91330, USA; 2 Department of Electrical Engineering, University of California, Los Angeles, California 90095, USA; 3 Inston Inc., Los Angeles, California 90095, USA

## Abstract

Voltage-induced switching of magnetization, as opposed to current-driven spin transfer torque switching, can lead to a new paradigm enabling ultralow-power and high density instant-on nonvolatile magnetoelectric random access memory (MeRAM). To date, however, a major bottleneck in optimizing the performance of MeRAM devices is the low voltage-controlled magnetic anisotropy (VCMA) efficiency (change of interfacial magnetic anisotropy energy per unit electric field) leading in turn to high switching energy and write voltage. In this work, employing *ab initio* electronic structure calculations, we show that epitaxial strain, which is ubiquitous in MeRAM heterostructures, gives rise to a rich variety of VCMA behavior with giant VCMA coefficient (~1800 fJ V^−1^m^−1^) in Au/FeCo/MgO junction. The heterostructure also exhibits a strain-induced spin-reorientation induced by a nonlinear magnetoelastic coupling. The results demonstrate that the VCMA behavior is universal and robust in magnetic junctions with heavy metal caps across the 5*d* transition metals and that an electric-field-driven magnetic switching at low voltage is achievable by design. These findings open interesting prospects for exploiting strain engineering to harvest higher efficiency VCMA for the next generation MeRAM devices.

## Introduction

Electric field (E-field) control of the magnetization vector via the magnetoelectric effect has sparked an explosion of technological and research interest due to its potential application in ultra-low power, highly-scalable, and non-volatile spin-based random access memory or MeRAM^1^,^2^,^3^,^4^. The realization of MeRAM is based on the voltage-controlled magnetic anisotropy (VCMA) of heavy-metal/ferromagnet/insulator (HM/FM/I) nano-junctions, where the non-magnetic HM contact electrode (Ta, Pd, Pt, Au) has strong spin-orbit coupling (SOC). In the linear regime, the VCMA is proportional to the E-field in the insulator, VCMA = *βE*_*I*_ = *βE*_*ext*_/*ε*_⊥_, where *β* is the VCMA coefficient, *E*_*ext*_ is the external E-field, and *ε*_⊥_ is the out-of-plane component of the relative dielectric constant tensor of the insulator. The challenge for achieving a switching energy per bit below that in complementary metal oxide semiconductor (~1fJ) and a write voltage below 1V requires large perpendicular magnetic anisotropy (PMA)^1^,^5^ and a VCMA coefficient higher than ~200 fJ V^−1^m^−1 ^6.

The VCMA of HM/FM/I junctions depends on the HM cap, the particular FM material or its alloys and exhibits a wide range of behavior ranging from linear to nonmonotonic ∨-shape or inverse-∨-shape (∧) E-field dependence with asymmetric *β*’s. A linear VCMA was observed in Ta/Co_40_Fe_40_B_20_/MgO^7^ and in Pd/FePd/MgO^8^ tunnel junctions with *β* of −33 and +600 fJV^−1^m^−1^, respectively, where the convention of positive E-field corresponds to electron accumulation at the FM/I interface. On the other hand, recent experiments on V/Fe/MgO revealed an asymmetric ∧-shape VCMA with giant *β* values of 1150 fJ V^−1^m^−1 ^9, while a ∨-shape VCMA was observed in double-barrier MgO/FeB/MgO/Fe junctions with *β* = 100 fJ V^−1^m^−1 ^10. Although in general the underlying mechanism of the giant *β* values remains unresolved, the internal E-field caused by charges trapped by defects in MgO can play an essential role^9^,^11^.

Similarly the VCMA of Au/FM/I trilayers with different FM materials remains unresolved and controversial. A linear VCMA was reported in Au/Fe_80_Co_20_/MgO junctions with *β* = −38 fJ V^−1^m^−1 ^12. Interestingly, Shiota *et al*. observed a voltage-induced magnetization switching from in- to out-of-plane direction^1^. In contrast, Au/Fe/MgO exhibits a ∨-shape VCMA^3^. On the theoretical side, *ab initio* electronic structure calculations of Fe/MgO^13^ and Au/Fe/MgO^14^ junctions with in-plane lattice constants of Fe and MgO, respectively, found a linear VCMA with *β* of about +130 and +70 fJ V^−1^m^−1^, respectively.

A ubiquitous feature in many HM/FM/I heterostructures is the large lateral strain among the HM, FM, and I components which can in turn tune the SOC and hence modify the magnetic anisotropy (MA) and magnetoelectric interfacial coupling. For example, nanostructures of AuCu/FeCo core-shell has been shown to possess superior strain-induced magnetic properties such as high saturation magnetization and coercivity^15^.

HM cap can considerably modify MA of magnetic layers^16^ and a combined strain and capping effect can strongly enhance VCMA of a HM/FM/I junction^17^. However, a direct E-field induced switching of magnetization is still elusive in both computation and experiments^3^,^7^,^8^,^9^,^10^,^13^,^14^,^17^,^18^,^19^. This raises further questions that (i) If the synergistic effect of strain and HM on VCMA is robust for cap materials across the 5*d* transition metals and (ii) Whether there exists a choice of HM which enables a direct E-field induced switching in a magnetic junction.

In this work we present *ab initio* electronic structure calculations which demonstrate that epitaxial strain has a strong effect on MA of Au/FeCo/MgO trilayer in nonlinear manner, leading to a strain-induced spin-reorientation. Furthermore, strain gives rise to a rich VCMA behavior ranging from a ∨-shape to inverse-∨-shape (∧) E-field dependence with *giant* coefficient. This demonstrates the universal and robust VCMA behavior in strained HM/FM/I junction for HM across the 5*d* transition metals. We also predict that an E-field-driven magnetization switching can be archived at low voltage.

### Effect of strain on zero-field MA

**Figure 1:**
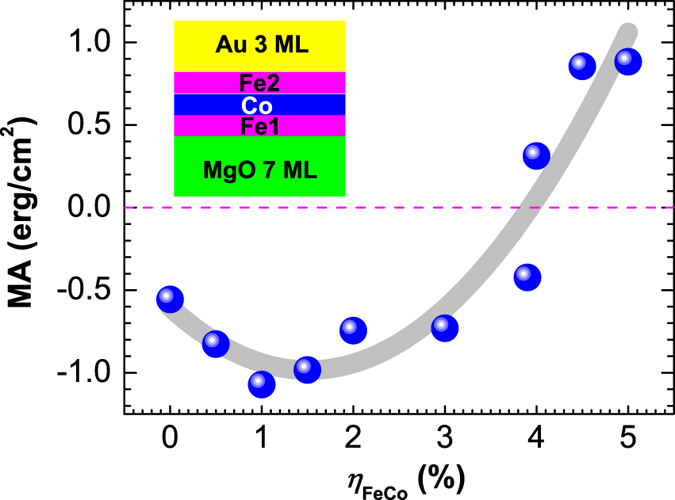
Strain dependence of zero-field MA where closed circles denote the *ab initio* results and the curve is a fit to equation (3).

Figure 1 shows the variation of the zero-field MA of the Au/FeCo/MgO junction with strain, *η*_*FeCo*_. The inset shows the schematic structure of Au/FeCo/MgO trilayer. The iron atoms at the Fe/MgO and Fe/Au interfaces are denoted by Fe1 and Fe2, respectively. The system shows a nonlinear magnetoelastic (MEL) behavior with a spin-reorientation at ~4% strain, in contrast to that in Ta/FeCo/MgO where MA is linearly dependent on strain with a magnetization switching occurs at ~2.5%^17^. This is a striking example on effect of HM cap on functional properties of a magnetic junction at nanoscale.

To explore the origin of the nonlinearity, we write the general expression MA = [*f*_*M*_(*e*_*n*_, *α*_1_ = 1) − *f*_*M*_(*e*_*n*_, *α*_3_ = 1)]*t*, where *t* is the FM thickness. The total magnetic energy-density functional of the FM, *f*_*M*_, is defined as


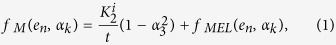


where *e*_*n*_ (*n* = 1–6) is strain tensor in Voigt notation, *α*_*k*_ (*k* = 1–3) is direction cosine of the magnetization vector, and 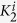 is the interface magnetocrystalline anisotropy. In a thin film grown epitaxially along the [001] crystallographic direction, there is no shear strain, *i.e., e*_5_ = *e*_5_ = *e*_6_ = 0. The MEL energy density *f*_*MEL*_ is expanded to second order^20^:


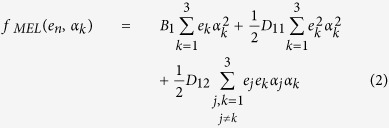


where *e*_1_ = *e*_2_ = *η*_*FeCo*_. The perpendicular strain *e*_3_ is then determined by minimizing the energy density *f* = *f*_*M*_ + *f*_*EL*_, where the elastic energy density 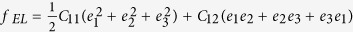, where *C*_*ij*_ is the elastic stiffness tensor with *C*_11_ ≈ 2*C*_12_ for transition metals^21^. The minimization gives rise to an MEL equation:


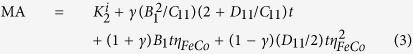


where 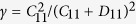.

For *D*_11_ = 0 or *γ* = 1, the nonlinear term in equation (3) identically vanishes. A finite *D*_11_ indicates that the presence of magnetization along a given direction induces an additional spontaneous strain on the FM film. Therefore, the combination of phenomenological analysis and *ab initio* calculation clearly shows that magnetic contribution to elastic behavior is significant and is the origin leading to the nonlinearity in the Au/FeCo/MgO trilayer. By fitting the *ab initio* results we find 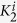 = −0.61 erg/cm^2^, *B*_1_ = −8.0 × 10^8^ erg/cm^3^ and *D*_11_ = 44.8 × 10^10^ erg/cm^3^. For bulk FeCo, the first-order MEL coefficient is 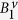 = −3 × 10^8^ erg/cm^3 ^22. From the expression 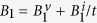, the interfacial contribution 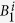 = −13.4 erg/cm^2^ can be inferred. For 100-nm-thick Fe film on MgO experiments reported *B*_1_ = −0.32 × 10^8^ erg/cm^3^ and *D*_11_ = 1.1 × 10^10^ erg/cm^3^. The value of *B*_1_ is close to that of bulk Fe of −0.34 × 10^8^ erg/cm^3^ since the surface and interface effects are small in a thick Fe film^23^. Our values for *B*_1_ and *D*_11_ are an order of magnitude larger than these values and are consistent with the fact that 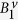 of bulk Fe_50_Co_50_ is an order of magnitude larger than bulk Fe. Moreover, in the Au/FeCo/MgO nano-juction interface effects are crucial.

**Figure 2:**
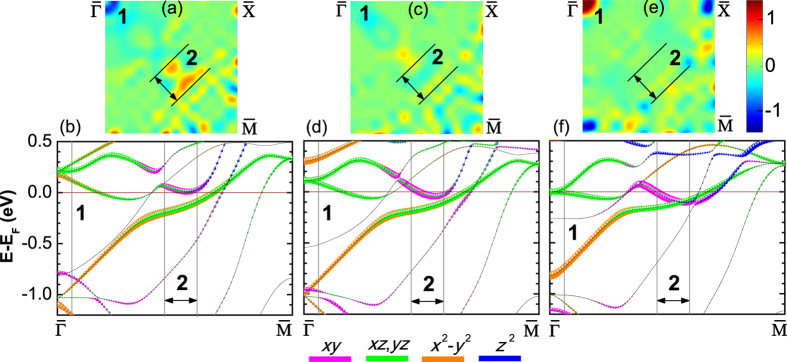
(**a**,**c**,**e**) *k*-resolved MA(**k**) (in meV) in the 2D BZ for *η*_*FeCo*_ = 0, 2 and 4%, respectively. (**b**,**d**,**f**) Energy- and *k*-resolved distributions of the orbital character of minority-spin bands along 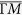 for the interfacial Fe1 atom *d*-states for *η*_*FeCo*_ = 0, 2 and 4%, respectively. Numerals in panels (**a**–**f**) refer to BZ **k**_||_ points (BZPn, n = 1, 2) where there are large changes of MA under strain.

To elucidate the microscopic mechanism of the strain effect on MA, we calculate *k*-resolved MA according to the force theorem^24^: MA(**k**) ≈ ∑_*n*∈*occ*_[*ε*(*n*, **k**)^[100]^ − *ε*(*n*, **k**)^[001]^] in the two-dimensional Brillouin zone (2D BZ). Here, *ε*(*n*, **k**)^[100]([001])^ are the eigenvalues of the Hamiltonian for magnetization along the [100] ([001]) direction. Figure 2(a,c,e) display MA(**k**) for *η*_*FeCo*_ = 0, 2 and 4%, respectively. Figure 2(b,d,f) show the corresponding energy- and *k*-resolved distribution of the orbital character of the minority-spin bands of the Fe1-derived *d*_*xy*_, *d*_*xz*,*yz*_, 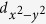, and 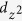 states along 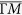 for *η*_*FeCo*_ = 0, 2, and 4%, respectively. We find that the strain-induced modification of the zero-field MA is mainly due to changes of the band structure of the interfacial Fe1 atom.

The MA can be expressed approximately in terms of the in- and out-of plane components of the orbital angular momentum operators 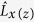 as^25^


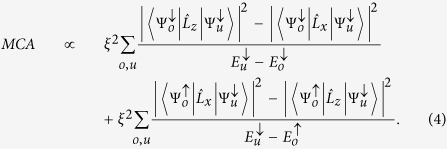


Here, 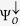

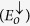
, 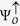 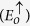, and 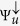 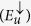 are occupied minority, occupied majority, and unoccupied minority-spin states (energies), and *ξ* is the SOC constant. Unless otherwise stated throughout the remainder of this manuscript the *d*-states refer to minority-spin states.

At zero strain, there is a minimum and a maximum of MA(**k**_**||**_) in Fig. 2(a) around 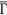 (BZP1) and 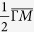 (BZP2), respectively. The negative MA at BZP1 around 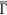 is due to the SOC of the minority-spin interfacial Fe1-derived occupied *d*_*xy*_ and 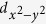 with the unoccupied *d*_*xz*,*yz*_, through the 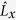 operator. On the other hand, at BZP2, there is strong SOC of the occupied Fe1 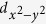
- and *d*_*xz*(*yz*)_-derived states close to the Fermi energy with the unoccupied *d*_*xy*_ and *d*_*yz*(*xz*)_ through the out-of-plane 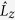, respectively. This in turn gives rise to a positive contribution to MA(**k**_**||**_), which, however, is partially compensated by the negative contribution from SOC of the occupied Fe1 *d*_*xz*,*yz*_ to the unoccupied *d*_*xy*_ and 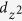 derived states [Fig. 2(b)].

At *η*_*FeCo*_ = 2% [Fig. 2(c,d)] there is a large downward shift of the occupied Fe1 *d*_*xy*_ around 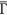 leading to an increase of its energy separation to the unoccupied Fe1 *d*_*xz*,*yz*_ states and hence an increase of MA(BZP1). At BZP2, the unoccupied *d*_*xy*_ and *d*_*xz*,*yz*_ slightly above the Fermi energy shift down and become occupied. This eliminates in turn the SOC of the occupied Fe1 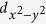
- and *d*_*xz*(*yz*)_-derived states with the unoccupied *d*_*xy*_ and *d*_*yz*(*xz*)_ through 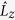, respectively. Consequently, the MA(BZP2) is reduced. Because the MA(**k**_**||**_) increases at BZP1 and decreases at BZP2, the total MA hardly changes upon increasing the strain from 0 to 2% (Fig. 1).

Under 4% strain the *k*-resolved MA, shown in Fig. 2(e), exhibits a sharp positive peak at BZP1 which is responsible for the magnetization vector switching from an in- to out-of-plane direction. The underlying origin in the electronic structure is that one of the Fe1 *d*_*xz*(*yz*)_ states at BZP1 slightly above the Fermi energy shifts downward and becomes occupied [Fig. 2(f)]. This in turn induces SOC of the occupied Fe1 *d*_*xz*(*yz*)_ with the unoccupied Fe1 *d*_*yz*(*xz*)_ around BZP1 through the out-of-plane orbital angular momentum operator 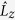.

### Effect of strain on VCMA

**Figure 3:**
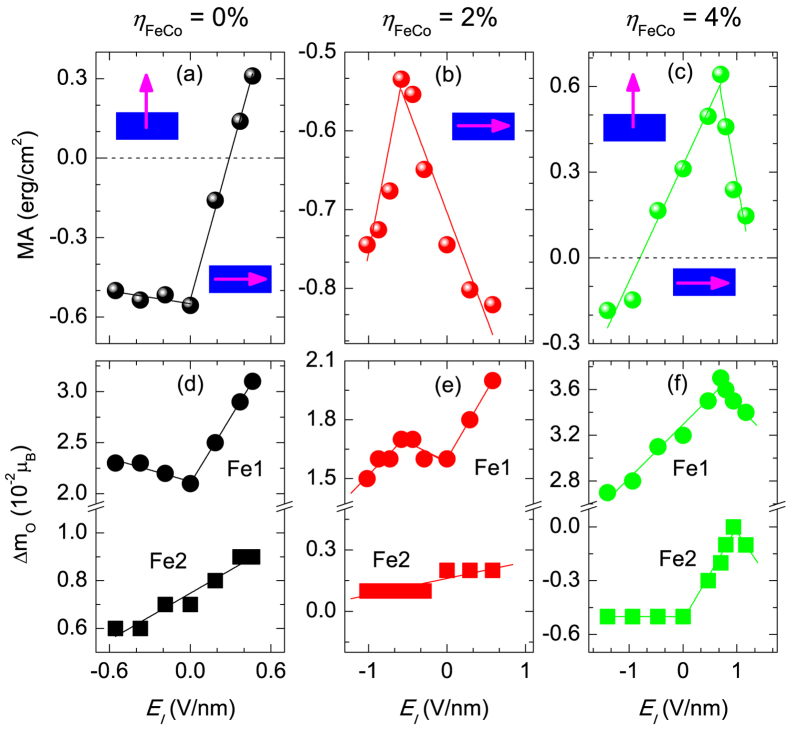
(**a**–**c**) MA versus E-field in MgO for different strain values. The vertical (horizonal) arrows indicate perpendicular (in-plane) magnetization. (**d**–**f**) Orbital moment difference, 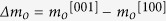, of the Fe1 and Fe2 interfacial atoms versus E-field for the same strain values.

The variation of MA as a function of the E-field in MgO is shown in Fig. 3(a–c) for *η*_*FeCo*_ = 0, 2 and 4%, respectively. The E-field in the insulator is inversely proportional to the *strain-dependent* out-of-plane component, *ε*_⊥_, of the dielectric tensor of the insulator. We find that *ε*_⊥_ increases exponentially with increasing compressive strain on the insulator (*i.e.*, decreasing expansive strain on the FM). The calculated values of the relative *ε*_⊥_/*ε*_0_ are 10.7, 17.0, and 27.0 for for *η*_*FeCo*_ = 4, 2, and 0%, respectively.

The results in Fig. 3(a–c) demonstrate that epitaxial strain gives rise to a wide range of intriguing VCMA behavior where the MA changes from (i) asymmetric ∨-shape field behavior under 0% strain with *β* values of 1871 (−101) fJ V^−1^m^−1^ for positive (negative) E-field; to (ii) asymmetric ∧-shape under 2% strain with *β* values of −246 (482) fJ V^−1^m^−1^ for E-field larger (smaller) than the critical field *E*_*c*_ = −0.58 V/nm where the MA reaches its maximum; and to (iii) asymmetric ∧-shape under 4% strain with *β* values of −1061 (393) fJ V^−1^m^−1^ for *E* ≥ (≤) *E*_*c*_ = 0.70 V/nm. Note that the range of *E*_*I*_ is below the breakdown field of MgO (~1 V/nm). In most experiments *E*_*I*_ is below 0.7 V/nm^4^,^7^, which is the value of *E*_*c*_ at 4%. Therefore, experimentally the VCMA appears linear at 4%. These VCMA coefficient values are the highest reported today and are larger by one to two order of magnitude compared to those reported in most experiments, except for those in refs 8,9 where charged defects may play a role.

More importantly, we predict an E-field-driven switching of the magnetic easy axis from in-plane to out-of-plane direction at 0.30 (−0.80) V/nm for *η*_*FeCo*_ = 0 (4)%. These findings have two important implications for magnetoelectric spintronics. First, the predicted VCMA coefficient values are very close to or larger than the critical value of ~200 fJ V^−1^m^−1^ required to achieve a switching bit energy below 1fJ in the next-generation of MeRAMs. Second, the results reveal the feasibility of tailoring the VCMA behavior via strain engineering to achieve desired MeRAM devices.

Figure 3(d–f) show the difference between the out-of- and in-plane orbital moments, 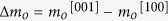, of the Fe1 and Fe2 interfacial atoms as a function of E-field for *η*_*FeCo*_ = 0, 2 and 4%, respectively. The E-field variation of Δ*m*_*o*_ for Co is much weaker and is not shown here. For single atomic species FMs with large exchange splitting the MA is related to the orbital magnetic moment anisotropy via the Bruno expression MA = *ξ*Δ*m*_*o*_/(4*μ*_*B*_)^26^. However, for structures consisting of multiple atomic species (as in the case of trilayers) with strong hybridization it has been shown that the expression is not satisfied and needs to be modified^27^. Overall the E-field dependence of Δ*m*_*o*_ for Fe1 and to a lesser degree of Fe2 correlates with that of the MA.

From the equation (4), E-field induced MA can originate from one or both of the following mechanisms: (i) charge screening or band filling effect, which is described by changes of the numerators and (ii) changes in separation of spin-orbit coupled pairs, which are described by the denominators. Thus far most of works assume either the band filling effect^13^,^28^,^29^,^30^ or the change in energy separation^14^,^31^ to be the predominant contribution. In the present work, we do not exclude possible contributions from the band filling effect. However, from our analysis the E-field induced changes in band structures can provide a consistent explanation of the E-field induced MA behavior, indicating that this is a plausible mechanism.

### VCMA at zero strain

**Figure 4:**
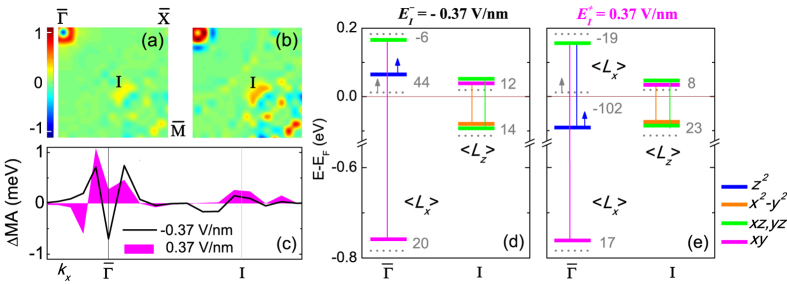
Zero strain: (**a**,**b**) E-field-induced change of MA, ΔMA(**k**), (in meV) in 2D BZ for 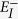 = −0.37 V/nm and 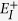 = 0.37 V/nm, respectively. (**c**) The E-field-induced ΔMA(**k**) along symmetry directions. (**d**,**e**) Fe1-derived electronic levels at the 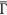 and I points (solid horizontal lines) under the negative and positive E-fields, where the numerals indicate shifts (in meV) of the energy levels compared to those at zero field (dotted lines). Majority-spin states are indicated by upward arrows and vertical lines connecting pairs of occupied and unoccupied states denote nonvanishing SOC matrix-elements where the line color matches that of the occupied state.

In order to understand the VCMA behavior under zero strain we show in Fig. 4(a,b) the E-field induced change of MA, ΔMA(**k**) = MA(**k**, *E*) - MA(**k**, *E*_*c*_ = 0), in the 2D BZ for 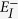 = −0.37 V/nm and 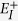 = 0.37 V/nm, respectively. Integration of the ΔMA(**k**) over the 2D BZ for negative and positive fields yields induced MA consistent with the asymmetric ∨-shape VCMA in Fig. 3(a). We also show in Fig. 4(c) the E-field induced ΔMA(**k**) along symmetry lines for positive and negative E-fields. Figure 4(d,e) display the E-field induced shifts of the energy levels (horizontal lines) of the Fe1-derived *d* states and the non-vanishing SOC matrix elements (vertical lines) between occupied and unoccupied *d* states at the 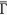 and I BZ points, where there are large changes of the MA.

The decrease of MA at 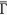 under negative E-field is due to the fact that the occupied minority-spin Fe1 *d*_*xy*_ shifts upward while the unoccupied minority-spin Fe1 *d*_*xz*,*yz*_ states shift downward. Consequently the energy separation of this pair of states, coupled via the in-plane orbital angular momentum, 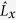, decreases resulting in a ΔMA(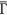) < 0. On the other hand, the increase of MA at 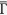 under positive E-field is associated with a large downward shift of the unoccupied majority-spin Fe1 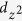 state to ~0.1 eV below the Fermi energy (Fig. 4(e)). This in turn induces a spin-mixed SOC between the majority-spin state and the unoccupied minority-spin *d*_*xz*,*yz*_ states via the in-plane orbital angular momentun 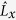 [second term in equation (4)], rendering ΔMA(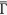) > 0.

At BZ point I under both negative and positive E-field the Fe1 minority-spin occupied *d*_*xz*,*yz*_ and 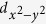 and unoccupied *d*_*xz*,*yz*_ and *d*_*xy*_ states shift upward resulting in a decrease of the energy separation between the occupied-unoccupied pairs by 2 meV and 15 meV, respectively. This leads to an increase of the MA contribution of the 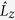 SOC matrix elements between (i) the occupied *d*_*xz*(*yz*)_ and unoccupied *d*_*yz*(*xz*)_ states and (ii) the occupied 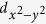 and unoccupied *d*_*xy*_ states, rendering ΔMA(**k**) > 0. The larger increase of ΔMA(**k**) at BZ point I under positive field correlates with the larger decrease of energy separation of the SOC pairs under positive E-field compared to that for negative E-field.

### VCMA at 2% strain

**Figure 5:**
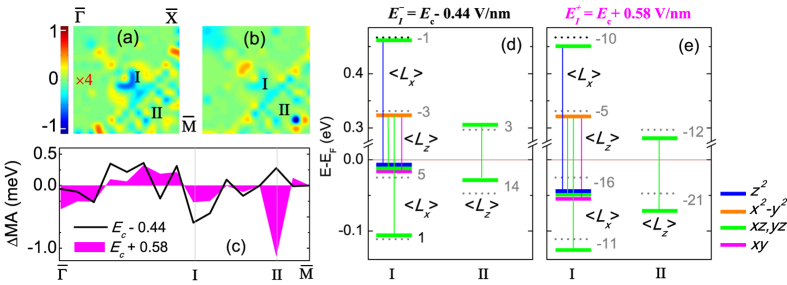
The same as Fig. 4(a–e) but for 2% strain and 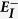 = *E*_*c*_ − 0.44 V/nm and 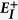 = *E*_*c*_ + 0.58 V/nm. The level shifts are with respect to those at the critical field *E*_*c*_ = −0.58 V/nm. For the sake of clarity, the contour values in (**a**) are magnified by 4 times.

Figure 5(a,b) show the E-field induced change of **k**-resolved MA, ΔMA(**k**) = MA(**k**, *E*)- MA(**k**, *E*_*c*_), in the 2D BZ for 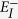 = *E*_*c*_ − 0.44 V/nm and 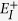 = *E*_*c*_ + 0.58 V/nm, where *E*_*c*_ = −0.58 V/nm is the critical E-field at 2% strain [Fig. 3(b)]. The contour values in Fig. 5(a) are magnified 4 times for the sake of clarity. Integration of the ΔMA(**k**) over the 2D BZ for 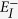 and 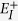 E-field yields induced MA consistent with the asymmetric ∨-shape VCMA in Fig. 3(b). The ΔMA(**k**) is plotted along symmetry lines in Fig. 5(c). In Fig. 5(d,e) we show the E-field induced shifts of the energy levels of the minority-spin Fe1-derived *d* states with respect to those at the critical field and the non-vanishing SOC matrix elements at the 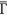, I and II BZ points, where there are significant changes of the MA.

Under 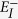, at the I BZ point the degenerate occupied Fe1 *d*_*xy*_, *d*_*xz*,*yz*_, and 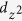 states near the Fermi level shift up while the unoccupied 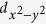 shifts down. This leads to an increase in the positive MA contribution of the 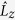 SOC matrix elements between the occupied Fe1 *d*_*xy*_ and unoccupied 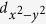 states. On the other hand, the E-field induced energy shifts decrease further the negative MA contribution of 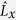 between (i) occupied Fe1 *d*_*xz*,*yz*_ with unoccupied 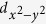 states and (ii) occupied 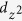 with the unoccupied *d*_*xz*,*yz*_ states. The interplay between the in- and out-of-plane orbital angular momentum matrix elements results in a *net* negative ΔMA(**k**). In sharp contrast the 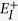 increases the separation between the occupied Fe1 *d*_*xy*_ and unoccupied 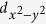 minority-spin states which are coupled through 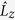. This in turn decreases the positive MA contribution of the SOC between these states rendering ΔMA(**k**) < 0.

At BZ point II ΔMA(**k**) is positive (negative) for 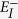 (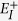) [Fig. 5(c)]. This is due to the decrease (increase) of the energy separation between the occupied Fe1 *d*_*xz*(*yz*)_- and unoccupied *d*_*yz*(*xz*)_-derived states, coupled through 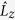, under 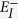 (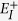) [Fig. 5(d,e)].

In summary, we have demonstrated that epitaxial strain, which is ubiquitous in many HM/FM/I trilayers, has a dramatic effect on the VCMA. It can change the VCMA from a ∨- to a ∧-shape E-field dependence with *giant* VCMA coefficients and tunable critical E-field. Furthermore, we have predicted that tuning of epitaxial strain can give rise to an E-field induced magnetization switching at low voltage. These result demonstrate that the universality and robustness of the VCMA behavior in strained HM/FM/I trilayers and that efficient E-field-driven magnetic switching can be attained by design. These findings open interesting prospects for exploiting strain engineering to harvest higher efficiency VCMA for the next generation MeRAM devices.

## Methods

The *ab initio* calculations have been carried out within the framework of the projector augmented-wave formalism^32^, as implemented in the Vienna *ab initio* simulation package (VASP)^33^,^34^. The generalized gradient approximation^35^ was employed to treat the exchange-correlation potential. To simulate the epitaxial growth of the Au/FeCo/MgO trilayer we employed a slab supercell along [001] consisting of three monolayers (MLs) of fcc Au, three MLs of *B*2-type FeCo, seven MLs of rock-salt MgO and a 15Å-thick vacuum region separating the periodic slabs. The 〈110〉 axis of MgO and Au are aligned with the 〈100〉 axis of FeCo and the O atoms at the FeCo/MgO interface are placed atop of Fe atoms. The iron atoms at the Fe/MgO and Fe/Au interfaces are denoted by Fe1 and Fe2, respectively [Fig. 1 inset]. Due to the large lattice constant mismatch between MgO and FeCo, the FeCo (MgO) is under expansive (compressive) strain, *η*_*FeCo*_ (*η*_*MgO*_), of ~+4% (−5.6%) compared with the lattice of bulk FeCo (MgO). Depending on the experimental conditions *η*_*FeCo*_ can vary from zero to 4%^36^. At each strain, the magnetic and electronic degrees of freedom and atomic *z* positions are relaxed *in the presence of the E-field* until the forces acting on the ions become less than ×10^−3 ^eV/Å and the change in the total energy between two ionic relaxation steps is smaller than 10^−6 ^eV. The plane-wave cutoff energy was set to 500 eV and a 15 × 15 × 1 Monkhorst-Pack *k*-mesh was used for the relaxation calculations. The SOC of the valence electrons is in turn included using the second-variation method^37^ employing the scalar-relativistic eigenfunctions of the valence states and a 31 × 31 × 1 k-point mesh. The MA per unit interfacial area, A, is determined from MA = [*E*_[100]_ − *E*_[001]_]/A, where *E*_[100]_ and *E*_[001]_ are the total energies with magnetization along the [100] and [001] directions, respectively.

## Additional Information

**How to cite this article**: Ong, P. V. *et al*. Electric-field-driven magnetization switching and nonlinear magnetoelasticity in Au/FeCo/MgO heterostructures. *Sci. Rep.* **6**, 29815; doi: 10.1038/srep29815 (2016).
